# Simultaneous Measurement of Glomerular Filtration Rate, Effective Renal Plasma Flow and Tubular Secretion in Different Poultry Species by Single Intravenous Bolus of Iohexol and Para-Aminohippuric Acid

**DOI:** 10.3390/ani10061027

**Published:** 2020-06-12

**Authors:** Lenka Stroobant, Siska Croubels, Laura Dhondt, Joske Millecam, Siegrid De Baere, Elke Gasthuys, Joachim Morrens, Gunther Antonissen

**Affiliations:** 1 Department of Pharmacology, Toxicology and Biochemistry, Faculty of Veterinary Medicine, Ghent University, Salisburylaan 133, 9820 Merelbeke, Belgium; lenka.stroobant@ugent.be (L.S.); siska.croubels@ugent.be (S.C.); laura.dhondt@ugent.be (L.D.); Joske.Millecam@poulpharm.be (J.M.); siegrid.debaere@ugent.be (S.D.B.); elke.gasthuys@ugent.be (E.G.); 2 Poulpharm bvba, 8870 Izegem, Belgium; 3 Flanders Institute for Biotechnology, Kapeldreef 75, 3001 Leuven, Belgium; joachim.morrens@kuleuven.be; 4 Interuniversity Microelectronics Centre (IMEC), Kapeldreef 75, 3001 Leuven, Belgium; 5 Department of Neuroscience, KU Leuven, Kapeldreef 75, 3001 Leuven, Belgium; 6 Neuroelectronics Research Flanders, Kapeldreef 75, 3001 Leuven, Belgium; 7 Department of Pathology, Bacteriology and Avian Diseases, Faculty of Veterinary Medicine, Ghent University, Salisburylaan 133, 9820 Merelbeke, Belgium

**Keywords:** effective renal plasma flow, glomerular filtration rate, iohexol, p-aminohippuric acid, poultry, renal physiology, tubular secretion

## Abstract

**Simple Summary:**

The aim of this study was to investigate the simultaneous measurement of two different renal markers (iohexol and p-aminohippuric acid) in the plasma of different poultry species as the gold standard method. The two markers reflect three different renal processes: glomerular filtration, effective renal plasma flow, and tubular secretion. The rate at which the kidneys filter blood is called the glomerular filtration rate. The effective renal plasma flow is the volume of plasma that reaches the kidney per time unit. Tubular secretion can be defined as active transport from the peritubular capillaries to the renal tubules. A moderate correlation was observed between tubular secretion and the glomerular filtration rate. A good correlation was demonstrated between the effective renal plasma flow and the glomerular filtration rate. This might be useful to model both renal processes. This approach could support the further development and validation of clinical renal biomarkers. These markers can be useful in the case of a chronic renal disease or renal failure, for which repeated evaluations of the renal function are required.

**Abstract:**

The aim of the current study was to investigate the simultaneous measurement of plasma p-aminohippuric acid (PAH) clearance as a potential marker to assess effective renal plasma flow (eRPF) and tubular secretion (TS), and the plasma clearance of iohexol (IOH) as a marker of the glomerular filtration rate in poultry species. The PAH was administered intravenously (IV) to broiler chickens, layers, turkeys, Muscovy ducks, and pigeons. Each animal received successively a single bolus dose of 10 mg PAH/kg bodyweight (BW) and 100 mg PAH/kg BW to assess the eRPF and TS, respectively. Simultaneously with both PAH administrations, a single IV bolus of 64.7 mg/kg BW of IOH was administered. A high linear correlation (R^2^ = 0.79) between eRPF, based on the clearance of the low dose of PAH, and BW was observed for the poultry species. The correlation between TS, based on the clearance of the high dose of PAH, and BW was moderate (R^2^ = 0.50). Finally, a moderate correlation (R^2^ = 0.68) was demonstrated between GFR and eRPF and between GFR and TS (R^2^ = 0.56). This presented pharmacokinetic approach of the simultaneous administration of IOH and PAH enabled a simultaneous evaluation of eRPF/TS and GFR, respectively, in different poultry species.

## 1. Introduction

Disease processes, diagnostics, and treatment modalities are subordinate to the physiologic and anatomic characteristics of the avian renal system [1]. In contrast to mammals, birds are characterized by a renal portal system; consequently, blood can flow to the kidneys along two ways, arterial and portal. Besides this, the cortical region of the avian kidney consists of two types of nephrons. The smallest or reptilian type consists of a proximal and a distal tubule without a loop of Henle. Due to the absence of a loop of Henle, these nephrons cannot contribute to the production of hyperosmotic urine. Next, there is a gradual transition to the deeper part of the cortex, which mainly consists of larger mammalian-type nephrons with a loop of Henle. The loops of Henle, collecting ducts, and vasa recta together form medullary cones, which mimic the medulla of the mammals [2]. The kidneys play a major role in the elimination of metabolic waste products (e.g., uric acid as main end product of the nitrogen metabolism), drugs, and toxic substances. Renal excretion is determined by the glomerular filtration rate (GFR), effective renal plasma flow (eRPF), tubular secretion (TS), and tubular reabsorption [3,4]. Consequently, methods analyzing these processes of renal excretion are essential in the clinical evaluation of renal disease and pharmacokinetic/pharmacodynamic integration in drug development and dosage-regimen optimization for veterinary medicine.

Interspecies drug dosage extrapolation depends on the similarity of the kinetic behavior of the drug or toxin in different species. Allometric scaling can provide a simple and fast option to interpolate and extrapolate dosages or pharmaco-/toxicokinetic parameters to a species of interest. This approach is a basic mathematical tool for analyzing differences in anatomy, physiology, biochemistry, and pharmaco-/toxicokinetics in animals of different sizes. Recently, a good correlation between the GFR and BW in different avian species has been demonstrated [5]. However, it is not known if the eRPF or TS obtained in one avian species might be extrapolated to other avian species.

The most well-known marker for the assessment of GFR is inulin, however iohexol (IOH), a radiographic contrast medium, has been proposed as an alternative marker in humans and several animal species [6]. IOH, a water soluble non-toxic monomer, is neither reabsorbed nor secreted by the renal tubules, with low hypertonicity as an additional advantage [3]. Recently, the clearance of an intravenous (IV) IOH bolus administration was applied as a sensitive marker to assess the GFR in six different bird species [5].

The clearance of p-aminohippuric acid (PAH) is considered to be the gold standard for the assessment of eRPF. PAH is a non-toxic organic anion which does not bind plasma proteins, and the erythrocyte membranes are not permeable for this molecule [3]. This compound undergoes extensive TS and negligible reabsorption. At low levels, such as 10 mg/kg bodyweight (BW) IV, this compound is almost completely cleared from plasma as it passes through the kidney. TS can be defined as the active transport of substances, such as endogenous organic compounds (e.g., bile salts and uric acid), drugs (e.g., some antimicrobial drugs, such as various penicillins and cephalosporins), and toxins (e.g., mycotoxin ochratoxin A) from the peritubular capillaries to the renal tubules [7,8,9,10,11]. TS is conducted by the proximal tubular epithelial cells by basolateral uptake and apical secretion into the tubule fluid using membrane transporters based on adenosine triphosphate (ATP) or transmembrane ion gradients [8]. In rats and dogs, studies have shown that administering a high dose of PAH (100 mg/kg BW) IV results in saturated active transport, which is a marker for TS [3].

Since IOH is only cleared by GFR, there is no interaction between IOH and PAH when they are simultaneously administered [12]. Hence, there is no competition at the level of the transporters between PAH and IOH for TS [3,12]. The simultaneous measurement of IOH and PAH would be a simple and reliable procedure which offers the opportunity to accurately determine the renal function with a limited sampling protocol [3]. A study of Rodriguez et al. has examined already the simultaneous determination of PAH and iopamidol in rats [3]. Laroute et al. studied GFR and eRPF in beagle dogs by the simultaneous measurement of iohexol and PAH [12]. However, no studies simultaneously determining the clearance of iohexol and PAH have been performed in birds.

Therefore, the aim of this study was first to investigate a pharmacokinetic approach for the simultaneous measurement of GFR and eRPF and GFR and TS based on the plasma clearance of IOH and PAH in different poultry species. Secondly, the aim was to apply allometric scaling to eRPF, TS, and GFR to evaluate whether these parameters can be extrapolated to other avian species.

## 2. Materials and Methods

### 2.1. Animal Trial

#### 2.1.1. Animals

The current study was in accordance with the national guidelines for the care and use of animals and was conducted with the consent of the Ethical Committee of the Faculty of Veterinary Medicine and Bioscience Engineering of Ghent University (EC2017/58). The experiment was carried out on broiler chickens (Ross 308, *Gallus gallus domesticus*, 6 weeks old, mean BW♂: 2.63 ± 0.46 kg, mean BW♀: 2.20 ± 0.37 kg), layers (Leghorn, *Gallus gallus domesticus*, 26 weeks old, mean BW♂: 1.79 ± 0.07 kg, mean BW♀: 1.47 ± 0.08 kg), turkeys (Hybrid Converter, *Meleagris gallopavo*,13 weeks old, mean BW♂: 12.68 ± 0.39 kg, mean BW♀: 9.38 ± 0.41 kg), Muscovy ducks (*Cairina moschata*, 6 months old, mean BW♂: 3.54 ± 0.16 kg, mean BW♀: 2.00 ± 0.06 kg), and pigeons (racing pigeons, *Columba livia forma domestica*, 6 months old, mean BW♂: 0.55 ± 0.01 kg, mean BW♀: 0.52 ± 0.04 kg), with eight birds (4♂/4♀) per species. All the birds were considered to be clinically healthy based on a physical examination and faecal examination prior and during the experiment and a macroscopic examination at necropsy after the experiment. All the animals were group housed per species in cages of 4–6 m^2^ in a temperature-controlled environment (18–25 °C), and the light cycle was 16 h light/8 h dark. The ducks had access to a pool of 1 m^2^. Water and feed were given to all birds ad libitum. The administered feed for turkeys, broiler chicken, layers, ducks, and pigeons was Dindo 2.3 pellet, Farm pellet 2, Gold 4 pellets, Duck 3 pellets, and Classic 4 seasons pigeons food, respectively (Versele-Laga, Deinze, Belgium).

#### 2.1.2. Experimental Design

Following a one-week acclimatization period, the animals were administered PAH (Sigma Aldrich, Bornem, Belgium) and a commercial IOH formulation (Omnipaque 300 mg I/mL, GE, Healthcare, Belgium). Immediately prior to administration, the PAH was dissolved in a physiological isotonic NaCl 0.9% solution at a final concentration of 100 mg PAH/mL and filtered through a polyethersulfone membrane syringe filter with a diameter of 13 mm and a pore size of 0.20 µm (Whatman, GE Healthcare, Buckinghamshire, UK). All the animals received PAH and IOH IV by cannulating the vena cutanea ulnaris superfacialis (wing vein) with a 25-gauge IV catheter (Terumo versatus winged IV catheter, Terumo Europe, Leuven, Belgium).

Two different dosages of PAH were tested in order to make a distinction between eRPF (low dose) and TS (high dose). On the first day of the study, all the birds received first a low IV dose of PAH (10 mg PAH/kg BW = PAH-LD). After administration, the IV catheters were flushed with an equal volume of 0.9% NaCl. Second, an IV bolus of IOH (64.7 mg/kg BW, 0.1 mL/kg BW Omnipaque^®^ 300 mg I/mL solution (GE Healthcare, Diegem, Belgium)) was administered. Before administration to the pigeons, the Omnipaque^®^ (GE Healthcare) solution was first diluted four times in 0.9% NaCl. After the IOH administration, the IV catheters were flushed again with an equal volume of 0.9% NaCl and subsequently removed. Blood samples (0.5 mL in chickens, ducks, and turkeys and 0.3 mL in pigeons) were taken from the vena metatarsalis plantaris superficialis and collected into heparin collection tubes at 0 (before administration), 5, 15, 30, 60, 120, 180, 360, 480, and 600 min post administration. The blood samples were transferred on ice and centrifuged for 10 min (2851× *g*, 4 °C) within two hours after collection. All the plasma samples were stored at ≤(−15) °C until further analysis.

After a three-day wash out period, the protocol was repeated with a high dose of PAH (100 mg/kg BW = PAH-HD) in combination with the same dose of IOH (64.7 mg/kg BW, 0.1 mL/kg BW Omnipaque^®^ 300 mg I/mL solution).

The plasma concentrations of iohexol and PAH were simultaneously determined by a previously in-house developed and validated ultra-high performance liquid chromatography–tandem mass spectrometry method [13]. The method was fully validated in broiler chicken plasma [13], followed by a more brief validation in the other avian species. Therefore, the within-day accuracy and precision were evaluated for IOH and PAH by analyzing 9 blank samples spiked at three different concentration levels: 1.0 (low), 10.0 (medium), and 75.0 (high) µg/mL on the same day. The acceptance criteria for accuracy were −20% to +10% for all the concentration levels. Within day precision were determined by calculating the residual standard deviation (RSD, %). The RSD value had to be below 10% for within-day precision. Corresponding species-specific matrix-matched calibration curves were used for the quantification of IOH and PAH in each sample (Table S1). A laboratory analysis was performed by operators who were blinded for animal species and PAH dosage.

### 2.2. Pharmacokinetic Analysis

A pharmacokinetic (PK) compartmental analysis was performed using Phoenix^®^ WinNonlin^®^ 6.4 (Certara, Cary, NC, USA). Plasma concentrations below the limit of quantification (LOQ) were excluded from the PK analysis. A two-compartmental model with first order elimination from the central compartment demonstrated the best fit for IOH and PAH high dose for all bird species. The low dose of PAH was best described with a one-compartmental model. The following PK parameters were calculated for low and high PAH doses: plasma concentration at time 0 (C_0_), area under the plasma concentration-time curve (AUC_0→inf_), elimination half-life (T_1/2el_), total body clearance (Cl), mean residence time (MRT), and volume of distribution (Vd). The plasma clearances of IOH and PAH were calculated by dividing the dose by the area under the plasma concentration time curve extrapolated to infinity (AUC_0→inf_).

### 2.3. Statistical Analysis

Statistical analyses were performed in R studio version 1.1.423. To take a correlation of within-animal datapoints into account, all the analyses using multiple datapoints per animal were performed using a mixed model. For other analyses, linear regression was performed. The level of significance was set at 0.05. For the analysis relating eRPF and TS, the missing values for those values were first imputed based on the linear regression equations relating eRPF to GFR and TS to GFR, respectively. The evaluation of the relationship between eRPF and TS was thus performed indirectly and was merely indicative, and the obtained *p*-values were not interpreted.

## 3. Results and Discussion

All the birds of the different poultry species survived the experiments without any complications. Table 1 summarizes the main PK parameters of the PAH and the total body clearance of IOH (CL_IOH_) when administered at the low dose (10 mg/kg) in combination with the nominal dose of IOH (64.7 mg/kg), respectively. Table 2 shows the main PK parameters of PAH and the CL_IOH_ when the high dose of PAH (100 mg/kg) was given in combination with the nominal dose of IOH (64.7 mg/kg).

Based on a simple linear mixed model, a highly significant (*p* < 0.001) relationship between GFR (=Cl_IOH_) and BW was demonstrated, expressed by the equation:

GFR (mL/min) = 4.11 + 1.59 × BW (kg) (R^2^ = 0.90) (1)

The GFR and BW were highly correlated, therefore it was decided to further analyze the impact of sex and species on the GFR/BW using mixed effects models. In a first model, the effects of species and sex including all the possible interactions were included, whereby none of the terms including sex were significant. Hence, a second mixed effects model was built only including species. Compared to the GFR/BW of broiler chickens (3.19 ± 0.08 mL/min/kg), the model identified a significantly lower GFR/BW in turkeys (1.90 ± 0.02 mL/min/kg; *p* = 0.008). In contrast, the GFR/BW was significantly higher in pigeons (6.70 ± 0.29 mL/min/kg; *p* < 0.001) and layers (5.01 ± 0.12 mL/min/kg; *p* < 0.001). Ducks also had a similar GFR/BW (3.58 ± 0.08 mL/min/kg) compared to broiler chickens, (*p* = 0.41). Similarly, a good correlation was observed between BW and GFR, based on the clearance of exo-IOH, in six different avian species. The results of the clearance of exo-IOH in the study of Gasthuys et al. were highly comparable to the results in this study for broilers, turkeys, and ducks (GFR exo-IOH (mL/min/kg) was: broiler chickens: 3.09; layer chicken: 2.57; turkey: 1.94; pigeon: 1.29; duck: 2.60; parrot: 1.11). However, a higher clearance of IOH was observed in this study for layers and pigeons [5]. Similarly, the GFR was 2.62–3.37 and 1.60–1.65 mL/min/kg in 18 weeks old layers and 4.5 weeks old broilers, respectively, based on inulin clearance [14,15]. Radin et al. observed a GFR of 3.12 mL/min/kg in layers based on the elimination of 3H-inulin following a single bolus injection [16]. The GFR estimated based on exogenous creatinine clearance in pigeons in the study of Scope et al. was similar (6.30 mL/min/kg) to the observed results in the presented study based on IOH clearance [17]. However, IOH was preferred to creatinine to measure the GFR in avian species, since it is not prone to tubular reabsorption nor secretion [5]. Furthermore, the sampling volume in pigeons was smaller in this study compared to the other poultry species, since collecting larger volumes of blood in smaller avian species is ethically not possible. Roberts et al. demonstrated the variability of the GFR values within the same bird species, which might explain the observed differences [18].

**Figure 1:**
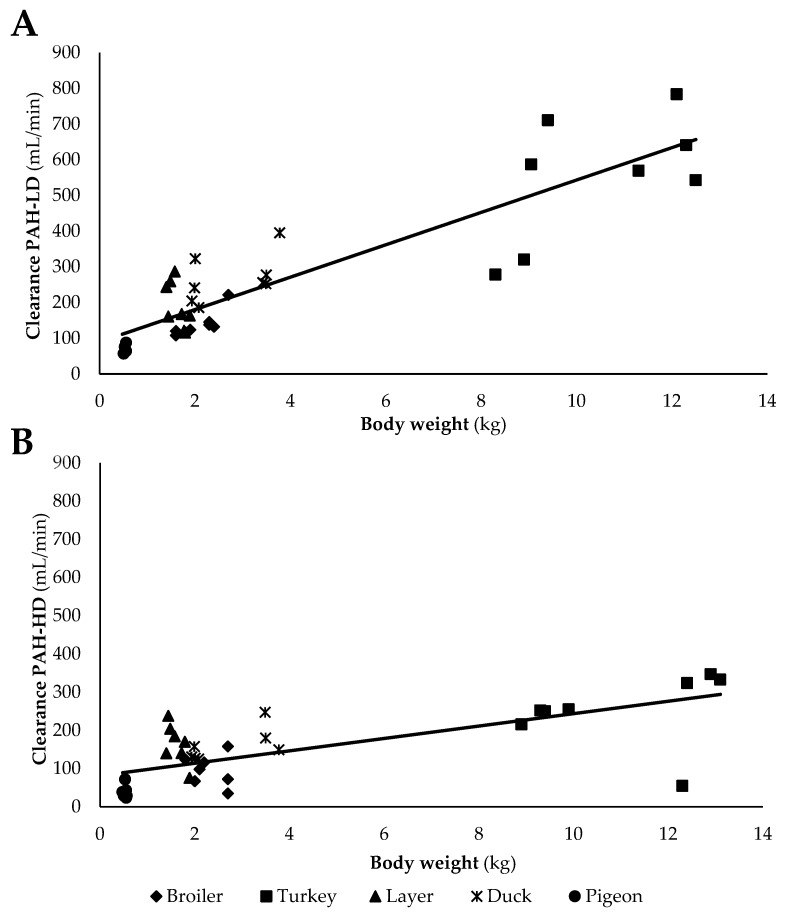
(**A**) Correlation between para-aminohippuric acid low dose (PAH-LD) clearance (mL/min) and body weight (kg). (**B**) Correlation between para-aminohippuric acid high dose (PAH-HD) clearance (mL/min) and body weight (kg) in different poultry species (broiler, turkey, layer, duck, and pigeon) (*n* = 8). The PAH-LD clearance is an estimate of the effective renal plasma flow, and the PAH-HD clearance is an estimate of tubular secretion.

Generally, the total body clearance of PAH (Cl_PAH_) per kg BW is higher in the group receiving the low dose of PAH (Table 1 and Table 2). In the present study, a high (0.70 < R^2^ < 0.90) linear correlation (R^2^ = 0.79) between Cl_PAH-LD_ and BW was observed for the different poultry species (Figure 1A), expressed by the formula:

eRPF = 89.72 + 45.32 × BW(2)

Figure 1B shows that the correlation between CL_PAH-HD_ and BW is less pronounced (R^2^ = 0.50), expressed by the formula:

TS = 81.61 + 16.26 × BW(3)

This correlation is considered as a moderate correlation (0.50 < R^2^ < 0.70). In contrast with the observed eRPF values of the different poultry species, a 5 to 12 times lower eRPF was demonstrated in dogs (13 mL/min/kg) [12]. However, compared to previous studies in layers, a higher eRPF was estimated based on the Cl_PAH-LD_ in this study. Radin et al. observed a CL_PAH_ of 22.81 mL/min/kg following IV administration of 14C-labelled PAH at a dose of 1.0 microCi to layers (male and female), compared to 79.44–160.88 mL/min/kg in the present study [16]. Similarly, Glahn et al.so observed a lower eRPF of 18 mL/min/kg in 18 week old layers based on the IV infusion of PAH (0.2–0.4 mg PAH/min/kg BW) [14]. In contrast to the observed PAH clearance of the different poultry species (20.74–130.32 mL/min/kg), a lower TS value has been observed (3.73 mL/min) in rats (BW 0.23–0.25 kg) [3].

**Figure 2:**
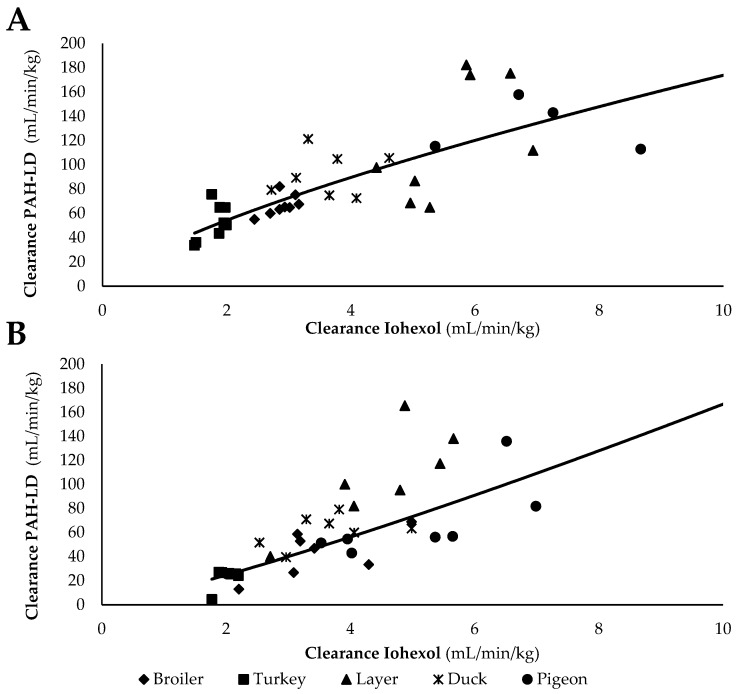
(**A**) Correlation between para-aminohippuric acid low dose (PAH-LD) clearance (mL/min/kg) and iohexol clearance (mL/min/kg). (**B**) Correlation between para-aminohippuric acid high dose (PAH-HD) clearance (mL/min/kg) and iohexol clearance (mL/min/kg) in different poultry species (broiler, turkey, layer, duck, and pigeon) (*n* = 8). The PAH-LD clearance is an estimate of the effective renal plasma flow, and the PAH-HD clearance is an estimate of tubular secretion. The iohexol clearance is a marker of the glomerular filtration rate.

A power regression model shows that there was a moderate correlation (R^2^ = 0.68) between the CL_PAH-LD_ (=eRPF) and the CL_IOH_ adjusted for BW (Figure 2A), expressed by the formula:

eRPF = 39.91 × GFR^0.72^ (4)

The relationship between the CL_PAH-HD_ (=TS) and the CL_IOH_ was also described by a power regression model: TS= 10.85 × GFR^1.19^ (R^2^ = 0.56; Figure 2B). Given, in the current study, that eRPF and TS could not be measured simultaneously, exploring the relationship between both variables cannot be done directly. The aforementioned relationships allow us to predict eRPF and TS respectively based on the GFR. Nevertheless, based on the fact that the relationships of GFR–eRPF and GFR–TS presented higher, the relationship between eRPF and TS can be estimated as follows:

TS = 0.02 × eRPF^1.65^ (5)

However, taking into account the only moderate correlation between GFR–eRPF and GFR–TS, this equation should be interpreted with some caution. The ratio clearance of PAH to the clearance of IOH is typically high when the given dose of PAH is low and vice versa. This indicates that a high dose of PAH is able to saturate the secretory system by a lower ratio. In contrast, Glahn et al. observed a higher clearance of PAH was observed in broiler chickens administered a dosage of 0.8 mg/min/kg compared to the period these birds received a dosage of 0.2 mg/min/kg by continuous infusion [15]. However, applied dosages were much lower compared to this study, also taking into account the short elimination half-life (T_1/2 el_) of PAH of only 0.09–0.20 h.

Although the presented approach of determining the eRPF and TS by administering an IV bolus of 10 or 100 mg PAH per kg BW, respectively, has already shown to be applicable in rats and dogs [3,12], future research is necessary to also prove the applicability in avian species by also evaluating birds with renal disease. Furthermore, the limited renal acetylation of PAH has been observed in humans but not in dogs [12,19]. Since metabolization affects the total clearance of the marker, the absence of metabolites in avian species would also needed to be confirmed.

A simultaneous administration of IOH and PAH, as markers for GFR and eRPF/TS, opens perspectives in small animal species research and medicine given the very sensitive method. Furthermore, the plasma IOH and PAH levels were simultaneously measured by ultra performance liquid chromatography - tandem mass spectrometer (UPLC-MS/MS). This study is based on the measurement of the markers in plasma instead of urine. The determination of the markers in urine is unreliable in birds given the coalesce of both urinary and gastrointestinal tract. Consequently, urine collection is difficult to perform in avian species [2]. An alternative to this approach is the determination of the total plasma clearance as a reflection of the renal clearance of the administered markers. This is only possible when a substance is solely renally cleared. In that situation, the renal clearance is equal to its plasma clearance. The main advantages of these procedures are the feasibility (single bolus administration in combination with repeated blood sampling over 10 h for this components) and the fact that there is no need for urine collection [12]. Furthermore, the presented approach requires only a single IV bolus injection PAH instead of a continuous infusion. Continuous infusion is difficult in animals, unless there is a chronically implanted IV catheter [12]. Furthermore, no adverse effects of simultaneous administration of IOH and PAH were observed in this study in healthy birds, but potential adverse effects on birds with renal dysfunction need further investigation. The simultaneous IV bolus administration of IOH and PAH should be taken into consideration for long-term studies when repeated evaluations of the renal function are required. Particularly to monitor for renal adverse effects in case of long-term treatment with potentially nephrotoxic drugs [3]. This simultaneous model opens perspectives for the further development of renal function tests.

## 4. Conclusions

In conclusion, a high correlation was demonstrated between the clearance of low dosage PAH and iohexol which might be used to model the eRPF in relation to the GFR, independent of bird species and sex. A moderate correlation was observed between the clearance of high-dosage PAH and iohexol. These findings can support the development and validation of biomarkers of renal function. Future research is necessary to evaluate the sensitivity, specificity, reproducibility, and safety of the presented approach in avian patients with renal insufficiency.

## Figures and Tables

**Table 1:** Main pharmacokinetic parameters of para-aminohippuric acid and iohexol clearance following the IV administration of 10 mg para-aminohippuric acid and 64.7 mg iohexol/kg bodyweight (BW) to different poultry species. Values (*n* = 8) are presented as mean ± SD.

Species	Sex	Iohexol	PAH-LD						
		Cl (mL/min)	Cl (mL/min)	Cl/BW (mL/min/kg)	Vd (L)	AUC_0→inf_ (h × µg/mL)	C_0_ (µg/mL)	T_1/2el_ (h)	MRT (h)
Turkey	♂	23.51 ± 0.68	643.1 ± 107.8	52.66 ± 8.88	9.51 ± 5.20	3.23 ± 0.52	15.17 ± 6.48	0.17 ± 0.06	0.24 ± 0.09
	♀	14.85 ± 2.35	474.2 ± 208.6	52.51 ± 20.96	5.58 ± 2.12	3.29 ± 1.11	16.83 ± 6.17	0.15 ± 0.07	0.21 ± 0.10
Duck	♂	12.63 ± 2.24	295.3 ± 67.6	82.86 ± 14.87	2.48 ± 1.05	2.05 ± 0.32	15.99 ± 5.69	0.09 ± 0.02	0.14 ± 0.03
	♀	7.34 ± 1.39	238.6 ± 60.75	119.3 ± 30.73	2.07 ± 0.88	1.46 ± 0.35	11.22 ± 5.15	0.10 ± 0.03	0.14 ± 0.04
Broiler chicken	♂	6.36 ± 1.13	156.4 ± 44.75	70.16 ± 10.34	2.34 ± 1.70	2.41 ± 0.35	12.32 ± 5.53	0.18 ± 0.14	0.26 ± 0.20
	♀	5.48 ± 0.34	120.2 ± 10.30	63.14 ± 5.51	1.84 ± 0.41	2.66 ± 0.25	10.54 ± 0.67	0.18 ± 0.03	0.25 ± 0.04
Laying chicken	♂	8.83 ± 0.89	142.3 ± 27.39	79.44 ± 15.46	1.31 ± 0.58	2.16 ± 0.41	15.64 ± 6.07	0.10 ± 0.03	0.15 ± 0.04
	♀	9.30 ± 0.75	237.8 ± 54.24	160.9 ± 32.90	2.39 ± 0.39	1.08 ± 0.28	6.27 ± 0.99	0.12 ± 0.01	0.17 ± 0.02
Pigeon	♂	3.13 ± 0.43	75.74 ± 16.68	136.5 ± 30.06	0.87 ± 0.45	1.25 ± 0.28	7.40 ± 3.86	0.13 ± 0.04	0.18 ± 0.06
	♀	4.58 ± 0.68	66.37 ± 13.24	127.9 ± 21.22	0.75 ± 0.75	1.32 ± 0.22	14.11 ± 14.38	0.15 ± 0.16	0.21 ± 0.23

IV, intravenous; BW, bodyweight; Cl, total body clearance; PAH-LD, para-aminohippuric acid low dose; Vd, volume of distribution; AUC_0_**_→_**_inf_, area under the plasma concentration time curve extrapolated to infinity; C_0_, plasma concentration at time 0; T_1/2el_, the elimination half-life; MRT, mean residence time.

**Table 2:** Main pharmacokinetic parameters of para-aminohippuric acid and iohexol clearance following the IV administration of 100 mg para-aminohippuric acid and 64.7 mg iohexol/kg BW to different poultry species. Values (*n* = 8) are presented as mean ± SD.

Species	Sex	Iohexol	PAH-HD						
		Cl (mL/min)	Cl (mL/min)	Cl/BW (mL/min/kg)	Vd (L)	AUC_0→inf_ (h × µg/mL)	C_0_ (µg/mL)	T_1/2el_ (h)	MRT (h)
Turkey	♂	24.82 ± 2.25	264.9 ± 140.3	20.74 ± 10.87	4.68 ± 4.08	141.2 ± 154.9	272.3 ± 192.9	0.16 ± 0.14	0.72 ± 0.51
	♀	19.05 ± 1.79	243.5 ± 18.55	25.96 ± 1.26	3.18 ± 1.22	64.33 ± 3.21	323.0 ± 104.4	0.15 ± 0.06	0.58 ± 0.53
Duck	♂	10.59 ± 1.18	192.47 ± 49.97	54.08 ± 15.81	4.50 ± 0.75	32.61 ± 9.28	80.93 ± 12.12	0.28 ± 0.10	0.82 ± 0.53
	♀	8.28 ± 1.28	135.1 ± 15.29	67.54 ± 8.39	2.07 ± 0.40	24.95 ± 2.90	99.97 ± 23.50	0.18 ± 0.05	0.65 ± 0.20
Broiler chicken	♂	9.14 ± 1.33	99.40 ± 51.19	39.72 ± 16.76	1.56 ± 1.32	46.67 ± 17.02	569.6 ± 772.0	0.20 ± 0.22	0.40 ± 0.22
	♀	7.28 ± 1.25	93.56 ± 40.29	45.45 ± 23.52	0.86 ± 0.54	54.66 ± 48.73	182.0 ± 32.88	0.09 ± 0.05	0.25 ± 0.03
Laying chicken	♂	6.82 ± 1.43	129.1 ± 48.54	72.47 ± 28.83	0.89 ± 0.35	26.46 ± 13.17	236.6 ± 130.2	0.08 ± 0.01	0.45 ± 0.08
	♀	7.36 ± 1.43	192.0 ± 41.01	130.3 ± 28.18	1.64 ± 0.62	13.24 ± 2.83	100.5 ± 38.06	0.10 ± 0.03	0.43 ± 0.21
Pigeon	♂	3.61 ± 2.35	31.90 ± 8.04	57.91 ± 14.77	0.56 ± 0.34	30.13 ± 7.20	144.5 ± 107.8	0.21 ± 0.15	1.03 ± 1.11
	♀	2.91 ± 0.66	42.11 ± 20.47	81.40 ± 38.63	0.41 ± 0.14	23.63 ± 9.15	144.1 ± 66.69	0.12 ± 0.03	0.52 ± 0.29

IV, intravenous; BW, bodyweight; Cl, total body clearance; PAH-HD, para-aminohippuric acid high dose; Vd, volume of distribution; AUC_0→inf_, area under the plasma concentration time curve extrapolated to infinity; C_0_, plasma concentration at time 0; T_1/2el_, the elimination half-life; MRT, mean residence time.

## Supplementary Materials

The following are available online at https://www.mdpi.com/2076-2615/10/6/1027/s1, Table S1: Detailed overview of within run accuracy and precision.
